# *Manilkara zapota* (L.) P. Royen Leaf Water Extract Induces Apoptosis in Human Hepatocellular Carcinoma (HepG2) Cells via ERK1/2/Akt1/JNK1 Signaling Pathways

**DOI:** 10.1155/2018/7826576

**Published:** 2018-11-05

**Authors:** Bee Ling Tan, Mohd Esa Norhaizan, Lee Chin Chan

**Affiliations:** ^1^Department of Nutrition and Dietetics, Faculty of Medicine and Health Sciences, Universiti Putra Malaysia, 43400 Serdang, Selangor, Malaysia; ^2^Laboratory of Molecular Biomedicine, Institute of Bioscience, Universiti Putra Malaysia, 43400 Serdang, Selangor, Malaysia; ^3^Research Centre of Excellent, Nutrition and Non-Communicable Diseases (NNCD), Faculty of Medicine and Health Sciences, Universiti Putra Malaysia, 43400 Serdang, Selangor, Malaysia; ^4^Department of Microbiology, Faculty of Biotechnology and Biomolecular Sciences, Universiti Putra Malaysia, 43400 Serdang, Selangor, Malaysia

## Abstract

*Manilkara zapota *(L.) P. Royen, called sapodilla, or locally known as* ciku*, belongs to the family* Sapotaceae*. We found that* Manilkara zapota* leaf water extract has cytotoxic effect against human hepatocellular carcinoma (HepG2) cell line in our earlier study. Therefore, this study aimed to explore the anticancer properties of* Manilkara zapota *leaf water extract in HepG2 cells. We also aimed to unravel yet undiscovered mechanisms and identified several expressed genes whose functions in cytotoxicity activity of* Manilkara zapota *leaf water extract in HepG2 cells have not been well-studied. The apoptosis and intracellular reactive oxygen species (ROS) activities were analyzed using Annexin V-propidium iodide staining and dichlorodihydrofluorescein diacetate, respectively, by NovoCyte Flow Cytometer. Bax and Bcl-2 expression were assessed using Enzyme-Linked Immunosorbent Assay. The associated molecular pathways were evaluated by quantitative real-time PCR. Overall analyses revealed that* Manilkara zapota* leaf water extract can increase percentage of early apoptotic cells, induce the formation of ROS, upregulate c-Jun N-terminal kinase 1 (*JNK1*) and inducible nitric oxide synthase (*iNOS*), and reduce* Akt1* and vascular endothelial growth factor A (*VEGFA*) transcriptional activities. Our data suggest that* Manilkara zapota* leaf water extract can suppress the growth of HepG2 cells via modulation of* ERK1/2*/*Akt1*/*JNK1 *transcriptional expression.

## 1. Introduction

Liver cancer has become the second most common cause of death worldwide and contributes to approximately 746,000 deaths in 2012 [1]. It represents the ninth leading cancer in women (228,000 cases) and the fifth in men (554,000 cases) [1]. Although tremendous efforts have been made in the last few decades to improve the current therapeutic approaches, conventional therapy is not likely effective due to an adverse outcome, yet metastasis and recurrence still tend to occur. Most of the anticancer drugs demonstrated a narrow therapeutic window with limited selectivity against cancer cells [2]. The use of systemic chemotherapy is hindered due to the chemoresistant in HepG2 cells, either extrinsic or intrinsic [3, 4]. Hence, the discovery of new anticancer agents from natural products has attracted an intense interest among scientists.

Mitogen-activated protein kinases (MAPKs), a family of threonine/serine protein kinases, are involved in the regulation of early apoptosis, which is crucial in several cellular processes, for example, cell adaptation and survival via phosphorylation of nuclear and cytoplasmic targets [5–8]. Inappropriate activation in MAPK signaling plays a crucial role in the progression and development of cancer [9]. Three distinct subgroups of MAPKs, namely, c-Jun N-terminal kinases (JNKs), protein kinase B (Akts), and extracellular signal-regulated kinases (ERKs), have been studied extensively [10]. Generally, JNKs are involved in the induction of apoptosis, while Akts and ERKs play a determining role in cell proliferation [11–13].

Inducible nitric oxide synthase (iNOS) has been associated with inflammation [14] by stimulating the expression of survival factors of cancer cells, thus suppressing the apoptosis. Apart from iNOS signaling, some of the well-known key angiogenesis and metastatic transcription factors described in* in vivo* study, including vascular endothelial growth factor A (VEGFA), have been demonstrated as potential therapeutic targets for cancer [15, 16]. In line with this, a prompt action is urgently needed to develop apoptosis-inducing agents with high efficacy and specificity but showing minimal undesirable effects.

*Manilkara zapota *(L.) P. Royen, generally known as sapodilla, or locally called* ciku *(family:* Sapotaceae*), is an evergreen tree found abundantly throughout Indian subcontinent, for example, in Bangladesh [17], although it is native to Central America and Mexico. This plant is traditionally used as folk medicine for the treatment of diarrhea and ameliorates pulmonary infections [18]. Relative proportions of bioactive components in* Manilkara zapota *leaf such as lupeol acetate, oleanolic acid, myricetin-3-O-*α*-L-rhamnoside, caffeic acid, and apigenin-7-O-*α*-L-rhamnoside have been reported by Fayek et al. [19], which are known to exhibit potent antioxidant activities [20]. The previous finding has demonstrated that* Manilkara zapota *leaf ethyl acetate extract inhibits the Ehrlich ascites carcinoma in mice [21]. Nevertheless, there is no pharmacological study on anti-liver cancer properties of* Manilkara zapota* leaf water extract in the literature. We found that leaf water extract of* Manilkara zapota* exhibited cytotoxic activity against human hepatocellular carcinoma (HepG2) cell line (unpublished data). Therefore,* Manilkara zapota* leaf water extract has a great potential to be developed as complementary and alternative medicine for the treatment of liver cancer. Nonetheless, the underlying mechanisms of* Manilkara zapota *leaf water extract inducing cytotoxicity in HepG2 cells require further elucidation. In view of the ability of* Manilkara zapota *leaf water extract to induce cytotoxicity in HepG2 cells, the anticancer properties of leaf water extract of* Manilkara zapota *in HepG2 cells were undertaken. We aimed to unravel yet undiscovered mechanisms and identified several expressed genes whose functions in cytotoxicity activity of* Manilkara zapota *leaf water extract in HepG2 cells have not been well-studied. These multiple genes may be involved in the regulation of hepatocellular cancer and deserve further study and discussion.

## 2. Materials and Methods

### 2.1. Chemicals and Reagents

Mycoplex™ fetal bovine serum (FBS), trypsin EDTA (1×), penicillin and streptomycin (100×), and RPMI-1640 medium were bought from Gibco (Grand Island, NY, USA). Annexin V-FITC Apoptosis Detection Kit I and Cycletest Plus DNA Reagent Kit were bought from BD Biosciences Pharmingen (Franklin Lakes, NJ, USA). Caspase Colorimetric assay kit was purchased from R&D Systems (Minneapolis, MN, USA). Bax and Bcl-2 Human SimpleStep ELISA® Kits were procured from Abcam, UK.

### 2.2. Cell Culture

The human hepatocellular carcinoma (HepG2) cell line was procured from American Type Culture Collection (ATCC; Rockville, MD, USA). The HepG2 cells were grown in RPMI-1640 medium supplemented with 10% (v/v) FBS, 100 *μ*g/mL streptomycin, and 100 IU/mL penicillin. The cells were grown at 5% CO_2_ atmosphere and 37°C humidified atmosphere incubator.

### 2.3. Plant Material

The plant (*Manilkara zapota *(L.) P. Royen) was collected from Pahang, Malaysia. The plant's authentication was conducted at Biodiversity Unit, Institute of Bioscience, Universiti Putra Malaysia (voucher specimen number: SK 3179/17).

### 2.4. Preparation of Plant Extract

Initially, leaf of* Manilkara zapota* was cut into small pieces and dried in an oven at 40°C for three days before being ground into powder form.* Manilkara zapota *leaf sample was extracted using water as previously reported by Tan et al. [22]. Five g of ground sample was extracted with 40 mL of water at 40°C for 2 h. The slurry was filtered using filter paper (Whatman No. 1) and the residues were reextracted. Lastly, the filtrate from water extract was freeze-dried using a freeze drier (Tecan, Switzerland) to obtain a concentrated powder.

### 2.5. Cell Viability Assay

Cytotoxicity of* Manilkara zapota* leaf water extract on HepG2 cells was evaluated using 3-(4,5-dimethylthiazol-2-yl)-2,5-diphenyltetrazolium bromide (MTT) assay [22]. Briefly, the HepG2 cells were seeded at a density of 5 × 10^4^ cells/well in a 96-well plate. After 24 h, the cells were treated with leaf water extract of* Manilkara zapota*. Untreated HepG2 cells (control) and 5-Fluorouracil (5-FU) (positive control) were also included. After treatment with* Manilkara zapota* leaf water extract for 24, 48, and 72 h, 20 *μ*L (5 mg/mL) of MTT was added to each well followed by incubation for 2-4 h. Active mitochondria in living cells reduced MTT to produce crystalline purple-blue formazan. After incubation for 2-4 h, media in each well were removed and 100 *μ*L of dimethyl sulfoxide (DMSO) was added to solubilize the purple-blue formazan. The absorbance was read at 570 nm using an ELISA microplate reader (Tecan, Switzerland), and 630 nm was used as a reference wavelength. Percentage of cell viability graph versus concentration of* Manilkara zapota* leaf water extract was plotted and the concentration of* Manilkara zapota* leaf water extract which inhibited 50% of cell viability compared to the control (50% inhibitory concentration (IC_50_)) was assessed. The cell viability was measured as follows:

(1) Percentage  of  cell  viability  %=OD570-630  treatmentOD570-630  control×100

where OD is the optical density.

### 2.6. Determination of Lactate Dehydrogenase Assay

Cytotoxicity was determined using an* in vitro *Toxicology Assay Kit by the release of lactate dehydrogenase (LDH), following the manufacturer's instruction. The cells were seeded at a density of 5 × 10^4^ cells in each well of 96-well plate. After an overnight incubation, the cells were exposed to different concentrations of* Manilkara zapota* leaf water extract for 24, 48, and 72 h, and the supernatant was collected and used to determine the LDH activity. The LDH mixtures were added to each sample in a volume equal to twice the volume of medium removed. The reaction was halted after addition of 1/10 (v/v) of 1 N HCl to each well and the absorbance was read at a wavelength of 490 nm using ELISA microplate reader (Tecan, Switzerland).

### 2.7. Determination of Cell Morphological Changes of Apoptosis

The HepG2 cells were seeded in each well of 6-well plate at a density of 1 × 10^5^ cells per well in 2 mL of complete growth medium. After 24 h incubation, the cells were exposed to 24, 48, and 96 *μ*g/mL of* Manilkara zapota* leaf water extract for 24, 48, and 72 h. Untreated cells (control) were also included. The morphological changes and the characteristics of apoptosis of the untreated HepG2 cells and HepG2 cells treated with* Manilkara zapota* leaf water extract were viewed under an inverted light microscope (Olympus, Center Valley, PA, USA).

### 2.8. Determination of Cell Cycle Arrest by Flow Cytometer

The Cycletest Plus DNA Reagent Kit was used to assess cell cycle arrest, according to the manufacturer's instruction. The HepG2 cells were seeded in 25 cm^2^ tissue culture flask at a density of 1 × 10^5^ cells and incubated for 24 h. The cells were exposed to 24, 48, and 96 *μ*g/mL* Manilkara zapota* leaf water extract for 24, 48, and 72 h. HepG2 cells were then centrifuged at 30 ×* g* for 5 min at room temperature followed by the addition of a buffer solution. The cells were then added with 250 *μ*L of solution A (trypsin buffer) and 200 *μ*L of solution B (RNase buffer and trypsin inhibitor), followed by 10 min incubation at room temperature, respectively. The mixture was mixed with cold solution C (200 *μ*L of PI stain solution) and allowed to incubate for 10 min at 4°C. Data acquisition and analysis were evaluated using NovoCyte Flow Cytometer (ACEA Biosciences, Inc.) with NovoExpress® software.

### 2.9. Determination of Apoptosis by Annexin V-Propidium Iodide Staining

The Annexin V-FITC Apoptosis Detection Kit I was used to analyze the activity of early and late apoptotic cells, according to the manufacturer's instruction. HepG2 cells were seeded in 25 cm^2^ tissue culture flask at a density of 1 × 10^6^ cells followed by an overnight incubation. The cells were exposed to 24, 48, and 96 *μ*g/mL of* Manilkara zapota* leaf water extract for 24, 48, and 72 h. After incubation with the respective time interval, the cells were trypsinized and rinsed twice with phosphate-buffered saline-bovine serum albumin-ethylenediaminetetraacetic acid (PBS-BSA-EDTA) and the cell pellet was resuspended in 100 *μ*L of 1 × binding buffer (0.1 M Hepes/NaOH, pH 7.4 and 1.4 M NaCl_2_, 25 mM CaCl_2_). An aliquot of 5 *μ*L of Annexin V-fluorescein isothiocyanate (FITC) and 10 *μ*L of propidium iodide (PI) were added to each sample and incubated for 10 min in the dark. Lastly, 400 *μ*L of 1 × binding buffer was mixed with the cells and the fluorescence was evaluated using a NovoCyte Flow Cytometer (ACEA Biosciences, Inc.) with NovoExpress® software.

### 2.10. Determination of Bax and Bcl-2 Activities in Manilkara zapota Leaf Water Extract

The Bax and Bcl-2 activities were quantified using Bax and Bcl-2 Human SimpleStep ELISA® Kits, according to the manufacturer's protocol. Initially, HepG2 cells were seeded in 25 cm^2^ tissue culture flask at a density of 1 × 10^5^ cells followed by an overnight incubation. The cells were treated with 24, 48, and 96 *μ*g/mL of* Manilkara zapota* leaf water extract for 72 h. The cells were trypsinized and centrifuged at 500 ×* g* for 5 min at 4°C to remove the medium. The cells were rinsed twice with phosphate-buffered saline (PBS) and cold 1× Cell Extraction Buffer PTR, followed by incubation on ice for 20 min. The cell lysates were subsequently centrifuged at 18,000 ×* g* and 4°C for 20 min, and the supernatants were collected. The protein concentrations were quantified using Bradford protein assay kit. An aliquot of the sample was diluted to the desired concentration in 1× Cell Extraction Buffer PTR. About 50 *μ*L of standard or sample was then added to 50 *μ*L of antibody cocktail in each well of 96-well plate. The plate was sealed prior to incubation for 1 h at room temperature on a plate shaker set to 400 ×* g*. Each well was rinsed with 3× 350 *μ*L 1× wash buffer PT. An aliquot of 100 *μ*L of TMB substrate was added to each well followed by 10 min incubation in the dark on a plate shaker set to 400 ×* g*. Subsequently, 100 *μ*L of Stop Solution was added to each well and read at the wavelength of 450 nm. Human Bax or Bcl-2 protein was used as a standard. The Bax standard stock solution (400 ng/mL) was prepared by adding 200 *μ*L of deionized water, followed by 10 min incubation. An aliquot of 225 *μ*L of 1× Cell Extraction Buffer PTR was added to tube number 1 and 150 *μ*L of 1× Cell Extraction Buffer PTR was added to tubes numbers 2-8. The stock solution was prepared using the dilution series. Standard tube number 8 contains no protein (blank control). The human Bcl-2 standard stock solution (200 ng/mL) was prepared by adding 1 mL of 1× Cell Extraction PTR incubated at room temperature for 3 min. Standards 2-8 were added with 150 *μ*L of 1× Cell Extraction Buffer PTR into each tube. A working dilution of Bcl-2 standard was prepared using a dilution series. Standard tube number 8 contains no protein (blank control).

### 2.11. Caspase-3 and Caspase-8 Assay

The caspase-3 and -8 activities were evaluated spectrophotometrically using a commercial colorimetric assay kit. Briefly, HepG2 cells were seeded in 6-well plate at a density of 1 × 10^5^ cells. After an overnight incubation, the cells were exposed to 24, 48, and 96 *μ*g/mL of* Manilkara zapota* leaf water extract for 72 h. The cells were trypsinized and centrifuged at 250 ×* g* for 10 min to discard the medium. The cell pellets were then lysed in 25 *μ*L of cold lysis buffer, followed by 10 min incubation on ice. The cell lysates were subsequently centrifuged at 10,000 ×* g* and 4°C for 1 min, and the supernatants were collected. The protein concentrations were quantified using Bradford protein assay kit. An aliquot of 50 *μ*L of 2 × Reaction Buffer 3 or 2 × Reaction Buffer 8 was mixed with 50 *μ*L of cell lysate containing 200 *μ*g of total protein, followed by 5 *μ*L of caspase-3 or caspase-8 colorimetric substrate (DEVD-*p*Na or IETD-*p*Na). Lastly, the reaction mixture was incubated at 37°C for 2 h before being read at a wavelength of 405 nm using ELISA microplate reader (Tecan, Switzerland).

### 2.12. Determination of Intracellular Reactive Oxygen Species in Manilkara zapota Leaf Water Extract

Dichlorodihydrofluorescein diacetate (DCFH-DA) was used to evaluate intracellular reactive oxygen species (ROS) in HepG2 cells treated with* Manilkara zapota* leaf water extract. Briefly, HepG2 cells were seeded in 6-well plate at a density of 1 × 10^5^ cells/well in 2 mL of complete media for overnight and pretreated with 10 *μ*M DCFH-DA in complete media for 1 h. The excess DCFH-DA was discarded and rinsed twice with PBS, followed by treatment with* Manilkara zapota* leaf water extract for 3 h. Following incubation, both adherent and floating cells were collected. The samples were then measured using NovoCyte Flow Cytometer (ACEA Biosciences, Inc.) with NovoExpress® software.

### 2.13. Determination of Antioxidants on Manilkara zapota Leaf Water Extract Induced Cell Death in HepG2 Cells

Briefly, the HepG2 cells were seeded at a density of 5 × 10^4^ cells/well in a 96-well plate, followed by an overnight incubation. The cells were treated with leaf water extract of* Manilkara zapota* or cotreated with 50 *μ*M *α*-tocopherol or ascorbic acid for 72 h. An aliquot of 20 *μ*L MTT (5 mg/mL) was added to each well followed by incubation for 2-4 h. The media in each well were removed and 100 *μ*L of DMSO was added to solubilize the purple-blue formazan. The absorbance was read at 570 nm using an ELISA microplate reader (Tecan, Switzerland), and 630 nm was used as a reference wavelength. The percentage of cell viability graph versus concentration of* Manilkara zapota* leaf water extract was plotted. The cell viability was measured as follows:

(2) Percentage of cell viability (%)=OD570-630treatmentOD570-630  control×100

where OD is the optical density.

### 2.14. Total RNA Extraction and Quantification

Total ribonucleic acid (RNA) was isolated using TRI Reagent®, according to the manufacturer's instruction. The HepG2 cells were seeded at a density of 1 × 10^5^ cells in a 25 cm^2^ culture flask for 24 h. After incubation for 72 h at different concentrations (24, 48, and 96 *μ*g/mL) of* Manilkara zapota* leaf water extract, the cells were homogenized and the lysates were aliquoted in microcentrifuge tubes. An aliquot of 1 mL TRI Reagent® was added in 25 cm^2^ tissue culture flask and resuspended. The homogenized sample was incubated for 5 min at room temperature to allow the dissociation of nuclear protein complexes. Hundred *μ*L of 1-bromo-3-chloropropane per mL of TRI Reagent® used was mixed and vortexed vigorously for 15 s followed by 2-15 min incubation at room temperature. After centrifugation for 15,000 ×* g* and 2-8°C for 15 min, the mixture was divided into a lower red organic layer, an interphase, and a colorless upper aqueous layer containing RNA. The aqueous layer was precipitated after the addition of 500 *μ*L of isopropanol. The sample was incubated for 5-10 min at room temperature prior to centrifugation at 11,500 ×* g* and 2-8°C for 10 min. The supernatant was discarded and the RNA pellet was washed with 1 mL of 75% (v/v) ethanol before being centrifuged at 5,500 ×* g* and 2-8°C for 5 min. Fifty *μ*L of RNase free water was mixed with the RNA pellet and resuspended before being stored at -80°C. The RNA concentration was read at 260 nm using a nanophotometer.

### 2.15. cDNA Synthesis

RNA sample was reverse-transcribed using the iScript™ gDNA Clear cDNA Synthesis Kit, according to the manufacturer's protocol. Briefly, 0.5 *μ*L of iScript DNase was added to 1.5 *μ*L of iScript DNase Buffer to make a DNase master mix. Two microliters of the DNase master mix was mixed with 14 *μ*L of RNA with an RNA amount of 2 *μ*g. The DNase reaction was conducted using a thermal cycler with the following mode and held at 4°C: 25°C for 5 min; 75°C for 5 min. The cDNA synthesis reaction mix was performed by adding 4 *μ*L of iScript Reverse Transcription Supermix and 16 *μ*L of DNase-treated RNA templates. The reverse transcription reaction was performed using an Authorized Thermal Cycler (Eppendorf, NY, USA) with the following conditions and held at 4°C: 25°C for 5 min; 46°C for 20 min; 95°C for 1 min.

### 2.16. Optimization of Primer Annealing Temperature

In order to optimize the annealing temperature of the designed primer sets, 2 *μ*L of cDNA (20 ng/*μ*L) was amplified using real-time polymerase chain reaction (PCR). Based on optimum annealing temperatures suggested by the manufacturer for the different primer sets, a gradient PCR program was conducted at different annealing temperatures ranging from 50.0°C to 63.0°C with 8 intervals (50.0°C, 50.8°C, 52.6°C, 55.1°C, 58.2°C, 60.8°C, 62.3°C, and 63.0°C) in CFX™ Real-Time System (Bio-Rad, Hercules, CA, USA).

### 2.17. Determination of Real-Time PCR Detection Limit and Primer Efficiency

To evaluate the detection limit and binding effect of the developed real-time PCR assay, a series of 10-fold dilution from undiluted sample to 10^−4^ of cDNA was prepared as a positive control while nuclease-free water was served as a non-template negative control. The detection limit of the assay was determined by analyzing the correlation between the concentration of the DNA template and Cq values as a standard curve.

### 2.18. Quantitative Real-Time Polymerase Chain Reaction (qRT-PCR)

Real-time PCR assay was performed using the designed primer sets with optimum annealing temperature determined from annealing temperature gradient analysis. Briefly, approximately 2 *μ*L of cDNA (20 ng/*μ*L) from* Manilkara zapota* leaf water extract was amplified using real-time PCR reaction using the designed primer sets originating from human cell lines (Table 1). PCR assay was conducted with the following conditions: 95.0°C for 2 min, followed by 39 cycles of 95°C for 5 sec, 55°C for 10 sec, and 72°C for 20 sec, before the fluorescence reading was recorded. The reactions were then incubated from 55.0°C to 95.0°C with 0.5°C increment per 10 sec for melt curve analysis. The fluorescence threshold limit of the CFX™ Real-Time System (Bio-Rad, Hercules, CA, USA) was set at 100 relative fluorescence units (RFU).

### 2.19. Statistical Analyses

Data are shown as means ± standard deviation with 3 independent analyses. The statistical significance of the difference between the control and treatment groups was analyzed using a one-way analysis of variance (ANOVA). Statistical analyses were performed using the Statistical Package for Social Science (SPSS) version 19.0 (SPSS Inc., Chicago, IL, USA). A* P* value less than 0.05 was considered statistically significant.

## 3. Results and Discussion

### 3.1. Manilkara zapota Leaf Water Extract Was Cytotoxic and Inhibits the Proliferation of HepG2 Cells

**Figure 1:**
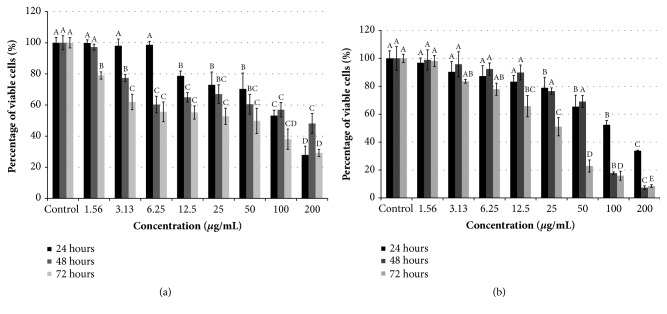
Cytotoxicity effect of* Manilkara zapota* leaf water extract on HepG2 cells. Cytotoxic effect of* Manilkara zapota* leaf water extract was evaluated on (a) MTT and (b) LDH assays. Values are reported as mean ± SD (n = 3). Value with different superscript letter indicates significant difference between groups by Tukey's test (*P *< 0.05). The percentage of viable cells was significantly inhibited at 24 h after treatment at higher concentrations (25-200 *μ*g/mL) compared to the untreated cells (control) in both MTT and LDH assays (*P *< 0.05). Treatment with 100-200 *μ*g/mL of* Manilkara zapota *leaf water extract for 48 and 72 h significantly reduced the cells viability in both MTT and LDH assays (*P *< 0.05). MTT: 3-(4,5-dimethylthiazol-2-yl)-2,5-diphenyltetrazolium bromide; LDH: lactate dehydrogenase.

Extensive histological and molecular evidence supporting the association between the apoptosis and anticancer activity of pharmaceutical agents has attracted many researchers to investigate new anticancer agents with potential apoptotic-inducing effect [23, 24]. Towards understanding the cells viability of* Manilkara zapota* leaf water extract on HepG2 cells, the apoptosis-inducing activity of* Manilkara zapota* leaf water extract was evaluated in HepG2 cells. According to published guidelines, any extract exerts potentially cytotoxic activities should have an IC_50_ less than 100 *μ*g/mL [25]. As presented in Figure 1(a), treatment with* Manilkara zapota* leaf water extract at higher concentrations (12.5-200 *μ*g/mL) for 24 h resulted in a significant reduction in the cell viability compared to the untreated cells (control) (*P* < 0.05). Consistent with the effects observed in 24 h, HepG2 cells also significantly reduced the cells viability after treatment with* Manilkara zapota* leaf water extract at 48 and 72 h compared to the control (*P* < 0.05), with the concentrations ranging from 3.13 to 200 *μ*g/mL. Prolong incubation time period up to 72 h caused the HepG2 cells to become more sensitive compared to 24 h (Figure 1(a)), with the IC_50_ value of 112.69 ± 6.51, 178.76 ± 8.57, and 48.24 ± 3.47 *μ*g/mL, respectively (unpublished data).

To address whether* Manilkara zapota* leaf water extract affected the proliferation of liver cancer cells, we treated HepG2 cells with different concentrations of* Manilkara zapota* leaf water extract and analyzed them using 3-(4,5-dimethylthiazol-2-yl)-2,5-diphenyltetrazolium bromide (MTT) and lactate dehydrogenase (LDH) leakage assays. In LDH assay, after irreversible cells membrane damage, a stable cytosolic enzyme of LDH that catalyzes the oxidation of _L_-lactate to pyruvate is released from the cytosol [26]. As shown in Figure 1(b), incubation* Manilkara zapota* leaf water extract for 24 h reduced the proliferation of HepG2 cells in a dose-dependent manner. A similar trend was also noted at 48 and 72 h. The IC_50_ values of* Manilkara zapota* leaf water extract towards HepG2 cells at 24, 48, and 72 h were 102.85 ± 7.96, 68.59 ± 9.14, and 49.07 ± 6.35 *μ*g/mL, respectively (unpublished data). Our LDH finding proved that* Manilkara zapota* leaf water extract was cytotoxic to HepG2, similar to the data shown from MTT proliferation assay, even though the findings of MTT assay exhibited a stronger cytotoxic effect on HepG2 cells. Based on the cytotoxic effect as evaluated by MTT and LDH assays, three concentrations (24, 48, and 96 *μ*g/mL) were selected for further analyses.

As a positive control, the HepG2 cells were incubated with the commercial drug, 5-Fluorouracil (5-FU). The IC_50_ values of 5-FU against HepG2 cells at 24, 48, and 72 h were 10.35 ± 4.79, 7.94 ± 2.91, and 2.55 ± 0.92 *μ*g/mL, respectively, as evaluated using MTT assay. Consistent with the MTT results, the findings from LDH assay also demonstrated that 5-FU suppresses the viability of HepG2 cells in a time-dependent manner, with IC_50_ values of 11.29 ± 4.94, 8.96 ± 3.52, and 3.08 ± 0.94 *μ*g/mL at 24, 48, and 72 h, respectively.

### 3.2. Morphological Changes of HepG2 Cells following Treatment with Manilkara zapota Leaf Water Extract

**Figure 2:**
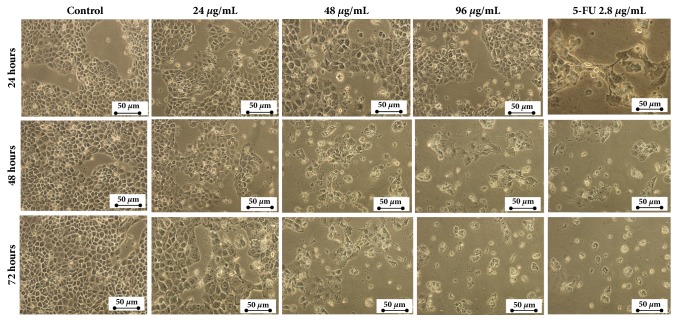
Morphological changes of HepG2 cells after treatment with* Manilkara zapota* leaf water extract (magnification 200 ×).* Manilkara zapota* leaf water extract inhibits the proliferation of HepG2 cells.

**Figure 3:**
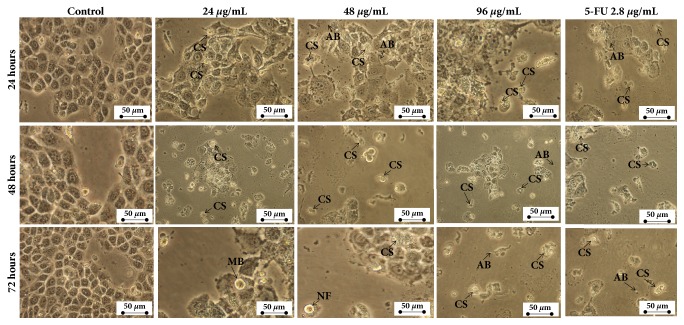
Close-up view of morphological changes in HepG2 cells after treatment with* Manilkara zapota *leaf water extract (magnification 400 ×) viewed under an inverted light microscope. The cells showed the apoptosis characteristics such as cellular shrinkage (CS), apoptotic bodies (AB), nuclear fragmentation (NF), and membrane blebbing (MB) (magnification 400 ×).

The morphological study revealed that* Manilkara zapota* leaf water extract induced growth inhibition and apoptosis in HepG2 cells. A marked inhibitory effect was also noted in HepG2 cells treated with 2.8 *μ*g/mL of 5-FU. As depicted in Figure 2, the number of cells in the group treated with 24 *μ*g/mL of* Manilkara zapota* leaf water extract was decreased compared to control from 24 h to 72 h. The growth of cells was inhibited after 48 h of incubation with 48 and 96 *μ*g/mL of* Manilkara zapota* leaf water extract and this phenomenon became more obvious at 72 h (Figure 2). Cell detachment was noted in HepG2 cells treated with 24 *μ*g/mL and in the latter (48 and 96 *μ*g/mL) from 24 h to 72 h. Notably, cell rounding and detachment of HepG2 cells were accompanied by an altered chromatin structure with a typical apoptotic morphology such as cellular shrinkage (CS), apoptotic bodies (AB), nuclear fragmentation (NF), and membrane blebbing (MB) (Figure 3).

### 3.3. Treatment with Manilkara zapota Leaf Water Extract Induces Cell Cycle Arrest in HepG2 Cells

**Figure 4:**
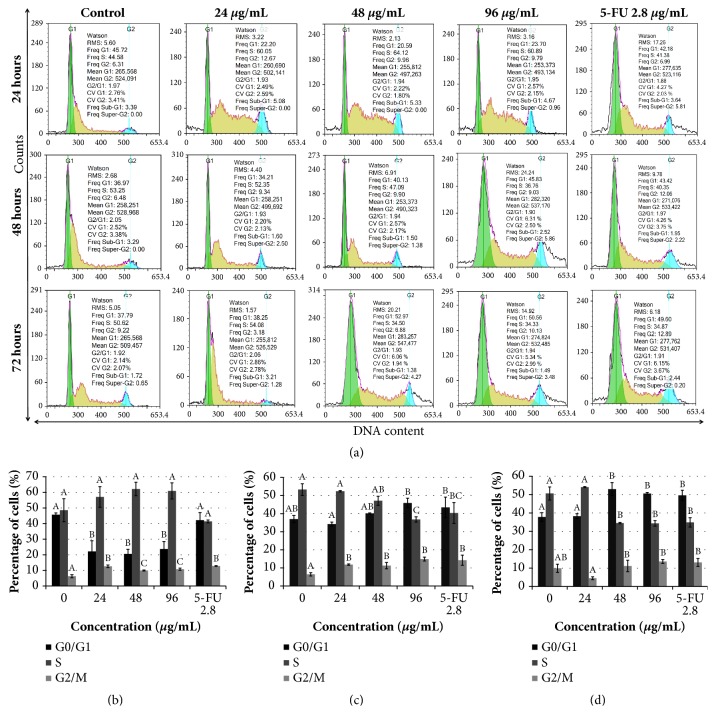
Cell cycle phase distribution of (a) untreated HepG2 cells and* Manilkara zapota *leaf water extract and 5-Fluorouracil (5-FU) treated HepG2 cells for (b) 24 h, (c) 48 h, and (d) 72 h, analyzed using flow cytometry. Values are reported as mean ± SD (n = 3). Value with different superscript letter indicates significant difference between groups by Tukey's test (*P* < 0.05). (b) A significant increase in the percentage of cells at G_2_/M phase was noted at 24, 48, and 96 *μ*g/mL of* Manilkara zapota *leaf water extract after 24 h (*P *< 0.05). (c) Treatment with* Manilkara zapota *leaf water extract at 24, 48, and 96 *μ*g/mL for 48 h significantly increased the population of cells at G_2_/M phase compared to the control (*P *< 0.05). (d) Treatment with* Manilkara zapota *leaf water extract at 48 and 96 *μ*g/mL significantly increased the population of cells at G_0_/G_1_ phase as compared to the control (*P *< 0.05) with a concomitant decrease of the S phase at 72 h.

Deregulation of the cell division process caused an uncontrolled proliferation and resulted in the development of tumor [27]. The ability to arrest cell cycle progression may serve as a potential anticancer agent [28]. To verify whether* Manilkara zapota* leaf water extract induced growth inhibition in HepG2 cells is modulated by cellular apoptosis and cell cycle arrest, the cells were incubated with different concentrations of* Manilkara zapota* leaf water extract for 24, 48, and 72 h and measured by flow cytometry (Figure 4(a)). Our analysis showed that a significant increase in the percentage of cells at G_2_/M phase was noted at 24, 48, and 96 *μ*g/mL of* Manilkara zapota* leaf water extract (*P *< 0.05) (Figure 4(b)). This finding indicates that* Manilkara zapota* leaf water extract elicited arrest at G_2_/M phase following 24 h of treatment at all tested concentrations (24, 48, and 96 *μ*g/mL).

As illustrated in Figure 4(c), treatment with* Manilkara zapota *leaf water extract at 24, 48, and 96 *μ*g/mL for 48 h significantly increased the population of cells at G_2_/M phase compared to the control (*P *< 0.05). In the present study, the treatment of* Manilkara zapota* leaf water extract elicited non-phase specific cell cycle arrest in HepG2 cells. On the other hand, treatment with* Manilkara zapota* leaf water extract at 48 and 96 *μ*g/mL significantly increased the population of cells at G_0_/G_1_ phase as compared to the control (*P* < 0.05) with a concomitant decrease of the S phase at 72 h (Figure 4(d)). The data we presented in this study demonstrated that* Manilkara zapota* leaf water extract destroys tumor cells in either dividing or resting state. The non-specific phase drug is considered as the most effective drug combating slow-growing tumors [29]. The mode of a non-phase specific antitumor agent is highly dependent on the incubation time and concentration [30]. This is also true for* Manilkara zapota* leaf water extract, whereby the growth inhibitory activity was observed in this study. Collectively, the data presented in this study suggest that the concentration and incubation time of* Manilkara zapota* leaf water extract may influence the effects on cell cycle.

### 3.4. Treatment with Manilkara zapota Leaf Water Extract Induces Apoptosis in HepG2 Cells

**Figure 5:**
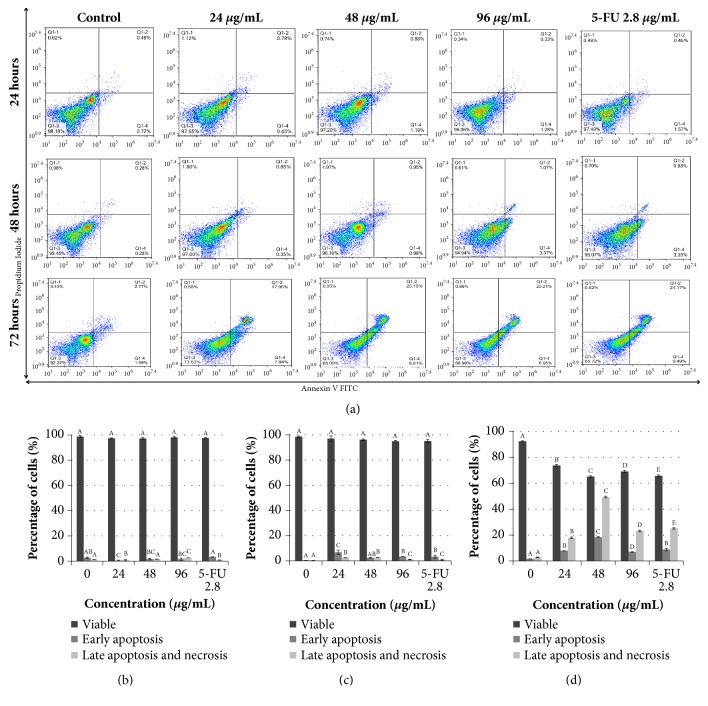
The percentage of viable, apoptotic, and necrotic cells of (a) untreated cells and* Manilkara zapota *leaf water extract and 5-Fluorouracil (5-FU) treated HepG2 cells for (b) 24 h, (c) 48 h, and (d) 72 h measured using the Annexin V-FITC and propidium iodide (PI) staining assay. Values are reported as mean ± SD (n = 3). Value with different superscript letter indicates significant difference between groups by Tukey's test (*P* < 0.05). (b) There was no significant difference in early apoptotic cells between control and 48 *μ*g/mL and 96 *μ*g/mL of* Manilkara zapota *leaf water extract (*P *> 0.05). The percentage of late apoptotic and necrotic cells after treatment with 96 *μ*g/mL (2.77%) of* Manilkara zapota* leaf water extract was significantly increased as compared to the control (1.31%) (*P* < 0.05). (c) Treatment with* Manilkara zapota *leaf water extract for 24 and 96 *μ*g/mL at 48 h significantly increased the percentage of early apoptotic cells as compared to the control (*P *< 0.05). The percentage of late apoptotic and necrosis cells also significantly elevated at 48 h, with a maximum effect observed at 24 and 48 *μ*g/mL as compared to the control (*P* < 0.05). (d) Treatment with* Manilkara zapota *leaf water extract for 72 h significantly increased the percentage of early apoptotic cells at 24, 48, and 96 *μ*g/mL (*P *< 0.05). The percentages of late apoptotic and necrosis cells were significantly increased after treatment with* Manilkara zapota* leaf water extract for 72 h (*P* < 0.05), with a maximum effect observed at 48 *μ*g/mL.

Apoptosis is a crucial mechanism in the cancer chemoprevention and chemotherapy [31, 32]. It acts as a silent cell death modality prior to the manifestation of malignancy [33]. Numerous chemotherapeutic drugs, including doxorubicin [34], tamoxifen [35], and cisplatin [36], inhibit neoplastic cells via cell cycle arrest and apoptosis induction. This suggests that natural products regardless of isolated bioactive compounds or crude extracts must stimulate the signals associated with cell death in order to serve as a potential agent in cancer therapy [37]. In order to validate* Manilkara zapota* leaf water extract induced apoptosis in HepG2 cells, Annexin V-FITC and propidium iodide fluorescence staining was measured quantitatively. The percentage of viable, early apoptotic cells, and late apoptotic and necrotic cells of untreated and* Manilkara zapota *leaf water extract treated HepG2 cells were measured by flow cytometry (Figure 5(a)).

Expectedly, we found no significant difference in early apoptotic cells between control and 48 *μ*g/mL and 96 *μ*g/mL of* Manilkara zapota* leaf water extract (*P* > 0.05). This result may reveal that the incubation time was too short to stimulate the early apoptotic cells (Figure 5(b)). Surprisingly, the percentage of late apoptotic and necrotic cells after treatment with 96 *μ*g/mL (2.77%) of* Manilkara zapota* leaf water extract was significantly increased as compared to the control (1.31%) (*P* < 0.05). The high percentage of late apoptotic and necrosis cells observed in this particular group of cells could be due to the incubation time being too short, where the release of intracellular content of HepG2 after cellular membrane damage has surpassed the antioxidant capacity [38]. Nevertheless, the increased percentage of late apoptotic and necrotic cells after treatment with 96 *μ*g/mL of* Manilkara zapota* leaf water extract in the present study remains to be elucidated.

Treatment with 24 and 96 *μ*g/mL of* Manilkara zapota* leaf water extract for 48 h significantly increased the percentage of early apoptotic HepG2 cells as compared to the control (*P* < 0.05). The percentage of late apoptotic and necrotic cells also significantly elevated at 48 h, with a maximum effect observed at 24 and 48 *μ*g/mL as compared to the control (*P* < 0.05) (Figure 5(c)). Notably, treatment with 2.8 *μ*g/mL of 5-FU significantly increased the early apoptotic cells as compared to the control (*P* < 0.05) (Figure 5(c)). Furthermore, our data revealed that treatment of HepG2 cells with* Manilkara zapota* leaf water extract for 72 h significantly increased the percentage of early apoptotic cells at 24, 48, and 96 *μ*g/mL for 7.84%, 18.30%, and 6.95%, respectively, as compared to the control (*P* < 0.05) (Figure 5(d)). This finding implied that* Manilkara zapota* leaf water extract might be used as a therapeutic agent for human liver cancer. Notably, the percentages of late apoptotic and necrotic cells were significantly increased after treatment with* Manilkara zapota* leaf water extract for 72 h (*P* < 0.05), with a maximum effect observed at 48 *μ*g/mL (Figure 5(d)). Based on the findings, leaf water extract of* Manilkara zapota *demonstrated the maximum apoptotic effect in HepG2 cells after 72 h. Therefore, an incubation time of 72 h was selected for further analyses.

### 3.5. Treatment with Manilkara zapota Leaf Water Extract Activates the Bax and Downregulates Bcl-2 Protein Levels in HepG2 Cells

**Figure 6:**
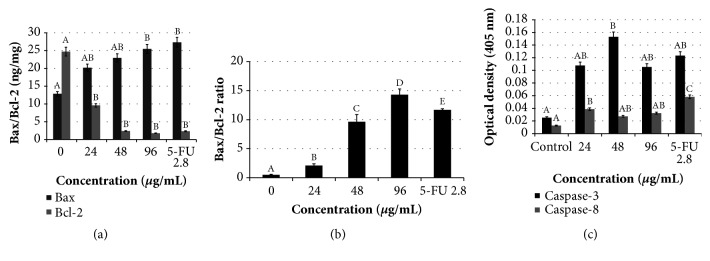
Apoptotic activities in HepG2 cells after treatment with* Manilkara zapota *leaf water extract and 5-Fluorouracil (5-FU) for 72 h. Apoptotic protein expression of (a) Bax and Bcl-2, (b) Bax/Bcl-2 ratio, and (c) Caspase-3 and -8 activities in HepG2 cells treated with* Manilkara zapota *leaf water extract. Values are reported as mean ± SD (n = 3). Value with different superscript letter indicates significant difference between groups by Tukey's test (*P* < 0.05). (a) Treatment with 96 *μ*g/mL of* Manilkara zapota *leaf water extract significantly upregulated Bax protein level (*p *< 0.05). However, there was no significant difference between 24 and 48 *μ*g/mL of* Manilkara zapota *leaf water extract (*P *> 0.05). Bcl-2 expression was significantly reduced after treatment with* Manilkara zapota *leaf water extract (*P *< 0.05). (b) Treatment of HepG2 cells with* Manilkara zapota *leaf water extract significantly induced a dose-dependent increase in the Bax/Bcl-2 ratio (*P *< 0.05). (c) The cells treated with* Manilkara zapota *leaf water extract at 48 *μ*g/mL significantly increase the caspase-3 activity compared with the control (untreated cells) (*P *< 0.05). Treatment with* Manilkara zapota *leaf water extract at 24 *μ*g/mL for 72 h also significantly upregulated the caspase-8 activity (*P *< 0.05). However, there was no significant difference in caspase-8 activity between 48 and 96 *μ*g/mL (*P *> 0.05).

Bcl-2 family proteins regulate the intrinsic mitochondrial pathway. The Bcl-2 family consists of the major apoptotic proteins which control the mitochondrial membrane permeability, such as antiapoptotic protein (Bcl-2) and proapoptotic protein (Bax) [39]. To explore the apoptotic protein expression and the underlying mechanism by which the* Manilkara zapota* leaf water extract induces apoptosis in HepG2 cells, Bax protein expression in HepG2 cells after induction with* Manilkara zapota* leaf water extract was evaluated. Our analysis revealed that treatment with 96 *μ*g/mL of* Manilkara zapota* leaf water extract significantly upregulated the Bax protein level (*P* < 0.05) (Figure 6(a)). Such a modulation may thus be involved in the proapoptotic effects of leaf water extract of* Manilkara zapota* and of bioactive constituents containing them. Taken together, the findings presented in this study suggested that* Manilkara zapota* leaf water extract initiates apoptosis in HepG2 cells through the mitochondrial intrinsic apoptotic pathway.

To confirm the apoptotic mechanisms induced by* Manilkara zapota* leaf water extract in HepG2 cells, Bcl-2 protein expression was assessed. Our data revealed that Bcl-2 protein expression was decreased in a dose-dependent manner, with a maximum reduction observed at a concentration of 96 *μ*g/mL (Figure 6(a)). This result was further supported by Tor et al. [40], who found that Bcl-2 expression was reduced after treatment with ethyl acetate extract of* Dillenia suffruticosa*. Overall, our findings confirm that leaf water extract of* Manilkara zapota* induces several subcellular mechanisms favoring apoptosis. In further analysis, we demonstrated that* Manilkara zapota* leaf water extract resulted in a dose-dependent increase in the Bax/Bcl-2 ratio (Figure 6(b)). Such upregulation of Bax and downregulation of Bcl-2 protein expression could lead to a major apoptotic response in HepG2 cells treated with leaf water extract of* Manilkara zapota*. Our data suggest that* Manilkara zapota* leaf water extract can induce apoptosis of HepG2 cells by regulating Bax/Bcl-2 ratio. Because our findings showed that the apoptotic response of HepG2 cells was modulated by Bax/Bcl-2 ratio, we further investigate the caspases-3 and -8 activities.

### 3.6. Treatment with Manilkara zapota Leaf Water Extract Promotes Activation of Caspase-3 and -8 Activities in HepG2 Cells

Numerous molecular studies have shown that two critical apoptotic pathways, which are intrinsically mediated mitochondrial and extrinsic modulated death receptor pathways, can be triggered by caspases [41]. Caspases are crucial molecular targets in chemoprevention because these processes contribute to apoptosis [42]. Thus, the activation of caspase-3 and -8 activities in HepG2 cells after exposure to* Manilkara zapota* leaf water extract was evaluated spectrophotometrically. To ascertain whether cell viability inhibition could be dependent on the stimulation of caspase-3 and -8, which serves as a central player in the modulation of apoptotic responses [43], the intracellular levels of caspase-3 and -8 in HepG2 cells after treatment with* Manilkara zapota* leaf water extract were investigated. As presented in Figure 6(c), the cells treated with* Manilkara zapota* leaf water extract at 48 *μ*g/mL significantly increased the caspase-3 activity compared with the control (untreated cells) (*P* < 0.05). In addition to the stimulation of caspase-3 activity, caspase-8 activity was also significantly upregulated after treatment with 24 *μ*g/mL of* Manilkara zapota* leaf water extract for 72 h (*P* < 0.05).

The quantification of caspase-3 and -8 enzymatic activities confirmed the caspase activation by leaf water extract of* Manilkara zapota*. Indeed, caspase-3 was activated at the median inhibition concentration (48 *μ*g/mL) of the treatment, while caspase-8 seems activated at the lowest concentration (24 *μ*g/mL) of the treatment. Collectively, this finding may suggest that an increase in caspase-8 activity results in the activation of the downstream apoptotic executioner caspase-3 and subsequently causes an activation of a molecular cascade of apoptosis in HepG2 cells. Taken together, our data indicate that* Manilkara zapota* leaf water extract inhibits the proliferation of liver cancer* in vitro*, which confirmed that the apoptosis induction by* Manilkara zapota* leaf water extract is caspase-mediated pathway.

### 3.7. Treatment with Manilkara zapota Leaf Water Extract Induces Reactive Oxygen Species Formation in HepG2 Cells

**Figure 7:**
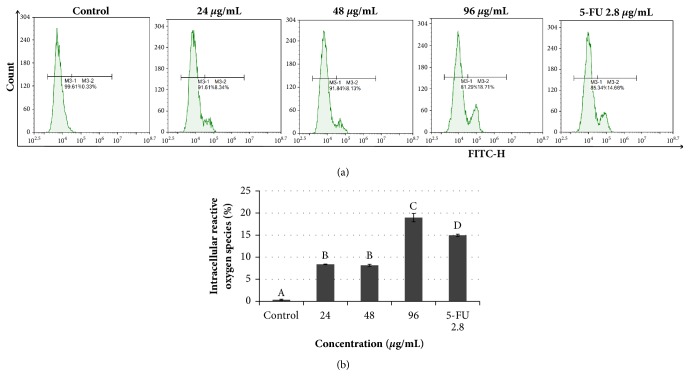
Determination of reactive oxygen species (ROS) in HepG2 cells. (a) Intracellular ROS in* Manilkara zapota *leaf water extract and 5-Fluorouracil (5-FU) treated HepG2 cells using dichlorodihydrofluorescein diacetate, measured using NovoCyte Flow Cytometer with NovoExpress® software. (b) Histogram presented the dichlorofluorescein (DCF) fluorescence intensity. Values are reported as mean ± SD (n = 3). Value with different superscript letter indicates significant difference between groups by Tukey's test (*P* < 0.05). Treatment with* Manilkara zapota *leaf water extract at a concentration of 24, 48, and 96 *μ*g/mL significantly elevated the ROS level compared to the control (*P < *0.05).

Previous studies have shown that biologically active compounds such as allicin combined with 5-Fluorouracil (5-FU) are involved in oxidative stress [44]. We speculated that* Manilkara zapota* leaf water extract may induce apoptosis via increasing reactive oxygen species (ROS). To validate this hypothesis, the fluorochrome dichlorodihydrofluorescein diacetate (DCFH-DA) was used to measure the ROS level by flow cytometry. Our data revealed that reactive oxygen species (ROS) level was mainly present in the groups treated with leaf water extract of* Manilkara zapota*, with a maximum effect observed at a concentration of 96 *μ*g/mL (Figures 7(a) and 7(b)). The observed effects were consistent with the results obtained by Cho et al. [45], who demonstrated that the elevation of ROS level triggers apoptosis in breast cancer cells. Further, chemotherapy agents have also been reported to induce oxidative stress and cause ROS generation [46]. Indeed, this finding indicates that excessive accumulation of ROS in the mitochondria may suppress the mitochondrial respiration chain and cause mitochondrial membrane rupture and apoptotic cell death [47, 48].

However, low ROS level was observed in untreated HepG2 cells (control). These data were further supported by Zou et al. [44], who found that low ROS level was mainly found in the untreated liver cancer (control). Importantly, this finding implies that low ROS levels may contribute to liver cancer, which contrasts its predicted role as a tumor suppressor [49].

**Figure 8:**
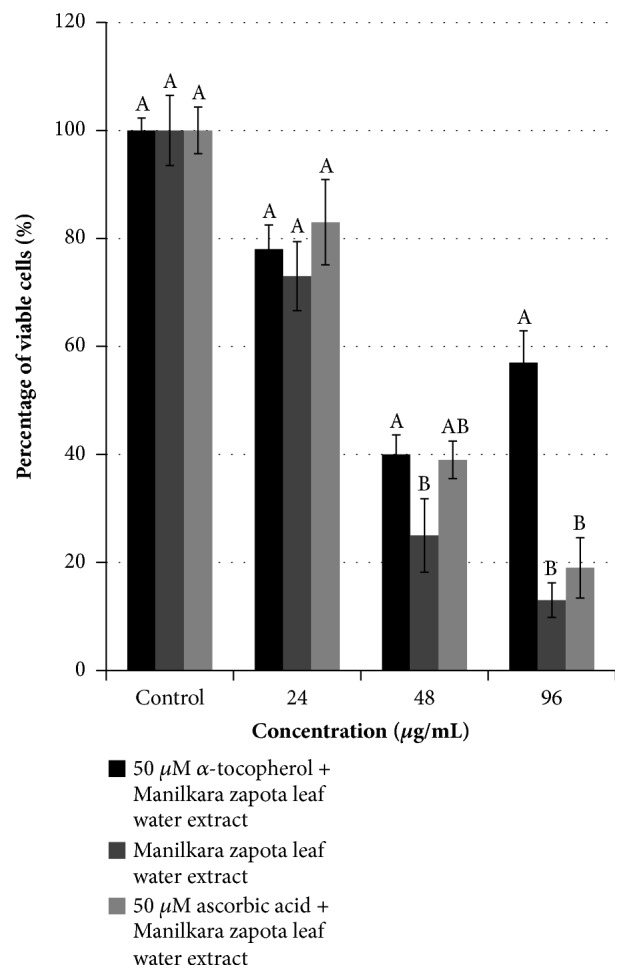
Determination on the involvement of ROS in* Manilkara zapota *leaf water extract in HepG2 cells. HepG2 cells were cotreated with 50 *μ*M *α*-tocopherol or ascorbic acid for 72 h. Values are reported as mean ± SD (n = 3). Value with different superscript letter indicates significant difference among the concentrations by Tukey's test (*P* < 0.05). At 96 *μ*g/mL, the cotreatment with 50 *μ*M antioxidant *α*-tocopherol and* Manilkara zapota *leaf water extract significantly increased the viability of the cells to 57% (*P* < 0.05). At 48 *μ*g/mL of* Manilkara zapota *leaf water extract, cotreatment with *α*-tocopherol significantly increased the viability of cells from 25% to 40% (*P* < 0.05).

To explore whether the cell death was mainly due to the ROS, the cells were cotreated with antioxidant ascorbic acid and *α*-tocopherol. As shown in Figure 8, the viability of the cells treated with* Manilkara zapota *leaf water extract alone at 96 *μ*g/mL for 72 h was 13%. The cotreatment with 50 *μ*M antioxidant *α*-tocopherol and* Manilkara zapota *leaf water extract significantly increased the viability of the cells to 57% (*P* < 0.05). At 48 *μ*g/mL of* Manilkara zapota *leaf water extract, cotreatment with *α*-tocopherol significantly increased the viability of cells from 25% to 40% ((*P* < 0.05). In comparison with* Manilkara zapota *leaf water extract alone, cells cotreated with 50 *μ*M ascorbic acid did not increase or decrease the viability of cells at all the tested concentrations (Figure 8).

Our present study demonstrated that ascorbic acid and *α*-tocopherol did not block the reduction of cell viability at 24 and 48 *μ*g/mL of* Manilkara zapota *leaf water extract, indicating that ROS may not be involved in the phenomenon at these two concentrations. However, at 96 *μ*g/mL of* Manilkara zapota *leaf water extract, the cotreatment with *α*-tocopherol significantly blocked the cell death, implying that high concentration of* Manilkara zapota *leaf water extract induced formation of ROS which played a central role in the induction of cell death in HepG2 cells. Taken together, these results suggest that a high concentration of* Manilkara zapota* leaf water extract induced the formation of ROS which played a crucial role in inducing cell death in HepG2 cells.

To gain a better understanding of this ROS-mediated apoptosis in HepG2 cells upon* Manilkara zapota* leaf water extract treatment, we evaluated the changes in the mRNA expression of extracellular signal-regulated kinase 1/2 (*ERK1/2*), protein kinase B (*Akt1*), c-Jun N-terminal kinase 1 (*JNK1*), inducible nitric oxide synthase (*iNOS*), and vascular endothelial growth factor A (*VEGFA*) using real-time polymerase chain reaction (PCR). Accumulating evidence suggests that ERKs and Akts have been implicated in cell proliferation [12, 50]. Therefore, we are interested in finding whether apoptosis induction of* Manilkara zapota* leaf water extract in HepG2 cells observed in this study could be modulated by ERK1/2 and Akt1 pathway. Based on the optimization, each gene of interest resulted in an efficiency of 89.6-108.7% (slope of -3.124 to -3.599). The melting curves analyses of the products demonstrated a single peak for each reference gene and target gene.

### 3.8. Treatment with Manilkara zapota Leaf Water Extract Inhibits ERK1/2 and Akt1 Response Pathway

**Figure 9:**
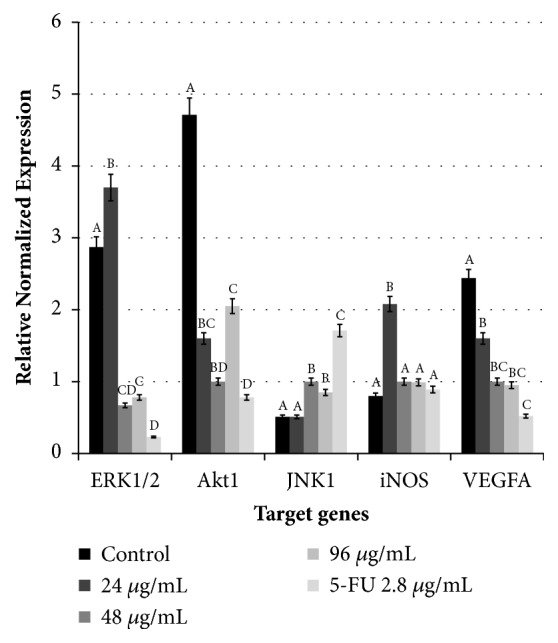
Expression of* ERK1/2*,* Akt1*,* JNK1*,* iNOS*, and* VEGFA *at mRNA levels in HepG2 cells incubated with* Manilkara zapota *leaf water extract and 5-Fluorouracil (5-FU) for 72 h analyzed using quantitative real-time PCR. Values are reported as mean ± SD (n = 3). Value with different superscript letter indicates significant difference between groups by Tukey's test (*P* < 0.05). The mRNA expression of* ERK1/2 *was significantly downregulated at 48 and 96 *μ*g/mL of* Manilkara zapota* leaf water extract treated HepG2 cells (*P *< 0.05).* Manilkara zapota *leaf water extract significantly upregulated the* ERK1/2 *mRNA level at 24 *μ*g/mL (*P *< 0.05).* Akt1* expression was significantly downregulated in HepG2 cells treated with 24, 48, and 96 *μ*g/mL of* Manilkara zapota *leaf water extract compared to the control (*P *< 0.05). Treatment with* Manilkara zapota *leaf water extract in a concentration of 48 and 96 *μ*g/mL significantly upregulated the* JNK1 *mRNA level (*P *< 0.05). Treatment with 24 *μ*g/mL of* Manilkara zapota *leaf water extract significantly upregulated the gene expression of* iNOS *(*P *< 0.05).* Manilkara zapota *leaf water extract significantly reduced the gene expression of* VEGFA *compared to the untreated cells (*P *< 0.05).* ACTB*: beta-actin;* ERK1/2*: extracellular signal-regulated kinase 1/2;* GAPDH*: glyceraldehyde-3-phosphate dehydrogenase;* iNOS*: inducible nitric oxide synthase;* JNK1*: c-Jun N-terminal kinase 1;* VEGFA*: vascular endothelial growth factor A.

Stimulation of ERK1/2 pathway is a crucial regulator in numerous cellular responses and cancer development [51]. To clarify the effects of* Manilkara zapota* leaf water extract in apoptosis of cancer cells, the expression levels of ERK1/2 were assessed. Real-time PCR analysis revealed that the mRNA expression of* ERK1/2* was significantly downregulated at 48 and 96 *μ*g/mL of* Manilkara zapota* leaf water extract treated HepG2 cells (*P* < 0.05) (Figure 9). These results suggest that treatment of HepG2 cells with* Manilkara zapota* leaf water extract may be associated with a marked increase in apoptotic cell death, as observed in Annexin V-FITC and propidium iodide fluorescence staining assay. Inhibition of* ERK1/2* mRNA level was also found in the reduction of the proliferation of breast cancer cells [52], which is consistent with the finding in this study. Therefore,* ERK1/2* mRNA level may play a crucial role in negatively regulating the ERK1/2 signaling pathway to inhibit the cell proliferation. Treatment with 48 and 96 *μ*g/mL of* Manilkara zapota* leaf water extract resulted in the downregulation of mRNA expression of* ERK1/2*. One of the possible reasons may be due to the efficiency of* Manilkara zapota* leaf water extract involved in the inhibition of* ERK1/2 *transcriptional activity reached with 48 and 96 *μ*g/mL.

However, upregulation of* ERK1/2* mRNA level was noted in 24 *μ*g/mL of* Manilkara zapota* leaf water extract (Figure 9), which is possibly associated with oxidative stress. Other than antioxidant defense, these effects also could be due to the cells counterbalance to the effect of oxidative stress via activation of the ERK1/2-dependent pathway. Collectively, our study suggests that* Manilkara zapota* leaf water extract could be a potent therapeutic agent against human hepatocellular carcinoma by phosphorylation of* ERK1/2* expression. Further studies of the interactions and the underlying mechanisms in* Manilkara zapota* leaf water extract induced apoptosis may pave the way to the knowledge of apoptotic network.

To further verify whether* Manilkara zapota* leaf water extract could suppress the proliferation of HepG2 cells, we determined the chemoprevention mechanism of* Akt1* on* Manilkara zapota* leaf water extract in this model. Akt is a serine-threonine kinase that controls the balance between apoptosis and survival.* Akt1 *expression was significantly downregulated in* Manilkara zapota* leaf water extract treated HepG2 cells compared to the control (*P* < 0.05), with a maximum reduction observed at a concentration of 48 *μ*g/mL (Figure 9), suggesting the involvement of Akt1 pathway in* Manilkara zapota* leaf water extract induced apoptosis. Research evidence found that apoptosis in cancer cells was associated with the suppression of Akt signaling pathway after treatment with retinoic-acid or Wogonin [53]. A similar finding was also reported by Huang et al. [54], who found that polyphenol derived from fruits and vegetables inhibited the migration of lung cancer via suppression of Akt activity. In line with the previous observations, our present study also demonstrated that* Manilkara zapota* leaf water extract downregulated the transcriptional activity of* Akt1*. Deregulation of Akt signaling pathway in cancer cells has become one of the therapeutic targets in the search of potential cancer treatment [55]. Therefore, this finding implies that* Manilkara zapota* leaf water extract has a potential in the treatment of liver cancer.

### 3.9. Treatment with Manilkara zapota Leaf Water Extract Induces JNK1 mRNA Level in HepG2 Cells

With regard to other transcription factors involved in cancer development, JNK is likely to be involved in coordination with oxidative stress. The expression of JNK is a stress-responsive kinase. Upregulation of JNK has been demonstrated to induce apoptosis in various cancers [56, 57]. The expression of JNK1 was abundantly present in the concentrations of 48 and 96 *μ*g/mL of* Manilkara zapota* leaf water extract (Figure 9). The activation of* JNK1* in the concentrations of 48 and 96 *μ*g/mL implied that higher concentration of bioactive components in the* Manilkara zapota *leaf water extract may confer better functional properties in the regulation of* JNK1*. This finding was consistent with the study reported by Palit et al. [58], who demonstrated that the expression of JNK was activated upon treatment with hesperetin, a flavanone glycoside predominantly contained in citrus fruit, subsequently triggering apoptosis. However, there was no significant difference in* JNK1 *expression between control and 24 *μ*g/mL of* Manilkara zapota *leaf water extract (*P *> 0.05). This result may reveal that the bioactive constituents present in this concentration are insufficient to stimulate the* JNK1*. Collectively, this study showed the transcriptional activation of* JNK1* following treatment with* Manilkara zapota* leaf water extract, suggesting that the activation of* JNK1* could be attributed to bioactive constituents present in* Manilkara zapota* leaf water extract (unpublished data), subsequently triggering apoptosis and inhibiting proliferation in HepG2 cells. Therefore, our results implied that there is an important link between transcriptional regulation and apoptosis modulation. Taken together, activation of* JNK1* mRNA level in* Manilkara zapota* leaf water extract treated HepG2 cells may play a crucial role in regulating the apoptosis.

### 3.10. Treatment with Manilkara zapota Leaf Water Extract Upregulates iNOS mRNA Level in HepG2 Cells

In addition to the effects observed in ERK1/2/Akt1/JNK1 signaling pathways, the roles of* iNOS* in the inhibition of liver cancer elicited by* Manilkara zapota* leaf water extract require further elucidation. Thus, the mRNA expression of* iNOS *in HepG2 cells was evaluated to determine whether* Manilkara zapota* leaf water extract could modulate the* iNOS *at the mRNA level. In the current study, we found a low mRNA expression of* iNOS* in the untreated HepG2 cells. The data presented in this study exhibited that treatment with 24 *μ*g/mL of* Manilkara zapota* leaf water extract significantly upregulated the gene expression of* iNOS* (*P* < 0.05) (Figure 9). The upregulation of* iNOS *mRNA expression in the present study was consistent with the findings reported by Radomski et al. [59], who observed that the expression of iNOS was negatively associated with metastasis in human-murine melanoma (K-1735) and colon cancer cells. Likewise, the study reported by Tan et al. [60] also found that* iNOS* overexpression inhibited the proliferation of colon tumor. Therefore, the upregulation of* iNOS* at the mRNA level may play a crucial role in the suppression of growth and induction of apoptosis in HepG2 cells. These findings suggest that* Manilkara zapota* leaf water extract reduced cancer proliferation via an anti-inflammatory mechanism involving* iNOS* expression. Notably, no significant difference was observed after treatment with 48 and 96 *μ*g/mL of* Manilkara zapota* leaf water extract (*P* > 0.05) (Figure 9). These findings demonstrated that* Manilkara zapota* leaf water extract did not block the reduction of cell viability at 48 and 96 *μ*g/mL in this signaling pathway.

### 3.11. Treatment with Manilkara zapota Leaf Water Extract Attenuates VEGFA mRNA Expression in HepG2 Cells

Because* Manilkara zapota* leaf water extract suppresses* ERK1/2* and* Akt1 *expression, the susceptibility of HepG2 cells to* Manilkara zapota* leaf water extract might also be due to the inhibition of metastasis via the suppression of* VEGFA* mRNA level. Thus, the mRNA level of* VEGFA* in* Manilkara zapota* leaf water extract was investigated to determine whether the extract could modulate* VEGFA* expression. The overall analysis indicated that untreated HepG2 cells presented the highest* VEGFA* expression compared with the groups treated with leaf water extract of* Manilkara zapota* (Figure 9). A significant reduction in the gene expression of* VEGFA *was also observed in the HepG2 cells treated with* Manilkara zapota* leaf water extract compared to the untreated cells (*P* < 0.05) (Figure 9). Our study revealed that treatment with* Manilkara zapota* leaf water extract resulted in the inhibition of* VEGFA* expression, and the maximum effect was obtained with 96 *μ*g/mL of* Manilkara zapota* leaf water extract. These data implied that* Manilkara zapota* leaf water extract has the potential to inhibit* VEGFA* expression. These findings indicate that* Manilkara zapota* leaf water extract might be involved in the suppression of metastasis or migration in HepG2 cells. The data presented in this study highlights the fact that* Manilkara zapota* leaf water extract contains potential antitumor and metastatic components for liver cancer cells. This result was consistent with the study obtained by Moyle et al. [61], who reported the potent inhibition of VEGF signaling by polyphenols. Such finding highlights the fundamental idea that polyphenol fraction can impact the angiogenic functions and inflammation. These data demonstrated that VEGFA is a key molecular target for* Manilkara zapota* leaf water extract which potently suppresses VEGF signaling and angiogenesis.

Phytochemical screening is one of the methods that have been employed to evaluate the antioxidant constituents in a plant sample. Of all phytochemicals, only flavonoids and saponins were present in* Manilkara zapota* leaf water extract in the qualitative analysis of phytochemicals. None of the steroids, triterpenoids, and phlobatannins was detected in the extract (unpublished data). The effects observed in this study could be due to the synergistic/additive effects of the phytochemicals such as flavonoids, saponins, and phenolic compounds [mainly gallic acid (23.11 ± 2.15 *μ*g/g), caffeic acid (3.04 ± 0.12 *μ*g/g), and vanillic acid (5.90 ± 0.71 *μ*g/g)] and antioxidant activity as evaluated using *β*-carotene bleaching test (49.94 ± 10.60%) and 1,1-diphenyl-2-picryl-hydrazyl (DPPH) radical scavenging capacity assays (0.24 ± 0.02 mg/mL) in the extract (unpublished data).

With regard to the present study, inhibition of the progression of liver cancer cells via multiple signaling pathways mediated apoptosis could be attributed by the presence of bioactive compounds in the* Manilkara zapota* leaf water extract. Importantly, our previous study also presented the safety of this extract in regard to the cell growth of noncancerous cells, such as mouse fibroblast (BALB/c 3T3) cell line (unpublished data).* Manilkara zapota *leaf water extract did not affect the cell viability of BALB/c 3T3 cell lines in the tested range, as the survival was consistently greater than 80% or similar to untreated BALB/c 3T3 cell lines. Therefore,* Manilkara zapota* leaf water extract might be a potential anticancer agent that can be used to suppress the development of liver cancer.

## 4. Conclusions

This study clearly showed that* Manilkara zapota* leaf water extract offers great potential against liver cancer via modulation of multiple signaling pathways. Our study demonstrated that* Manilkara zapota* leaf water extract upregulates* JNK1* and* iNOS* and transcriptional downregulation of* ERK1/2*,* Akt1*, and* VEGFA* expression implies the potential use of* Manilkara zapota* leaf water extract in future applications to combat liver cancer. However, to fully elucidate the potential of* Manilkara zapota* leaf water extract as an anticancer agent, further in-depth studies such as animal experimentation are needed to provide valuable insights to develop it as a therapeutic approach for the treatment of human hepatocellular carcinoma and other human malignancies. Altogether, this finding provides substantial evidence that* Manilkara zapota* leaf water extract induces early apoptosis in HepG2 cells via modulation of intrinsic mitochondrial pathways and suppression of metastasis. Taken together, our data suggested that* Manilkara zapota* leaf water extract has noteworthy apoptotic potentials via the regulation of* ERK1/2*/*Akt1*/*JNK1* transcriptional activity.

## Figures and Tables

**Table 1:** Nucleotide sequence of PCR primers for amplification and sequence-specific detection of cDNA (obtained from the GenBank database).

Primer name [accession number]	Oligonucleotides (5′-3′) sequence
*ERK1/2 * [NM_002745.4]	F– CCACCCATATCTGGAGCAGT R– CAGTCCTCTGAGCCCTTGTC
*Akt1 *[*AB*451242]	F– AGAAGCAGGAGGAGGAGGAG R– TCTCCTTCACCAGGATCACC
*JNK1 * [NM_139046.3]	F– GTGATCAATGGCTCTCAGCA R– TGACTAACCGACTCCCCATC
*iNOS *[AF049656.1]	F– GTGGTGACAAGCACATTTGG R– GTCATGAGCAAAGGCACAGA
*VEGFA * [NM_001287044.1]	F– CCCACTGAGGAGTCCAACAT R– AAATGCTTTCTCCGCTCTGA
*ACTB* ^a^ [NM_001101.3]	F– AGAGCTACGAGCTGCCTGAC R– AGCACTGTGTTGGCGTACAG
*GAPDH* ^a^ [NM_002046.4]	F– GGATTTGGTCGTATTGGGC R– TGGAAGATGGTGATGGGATT
18S rRNA^a^ [HQ387008.1]	F– GTAACCCGTTGAACCCCATT R– CCATCCAATCGGTAGTAGCG

*ACTB*: beta-actin; *ERK1/2*: extracellular signal-regulated kinase 1/2; *GAPDH*: glyceraldehyde-3-phosphate dehydrogenase; *iNOS*: inducible nitric oxide synthase; *JNK1*: c-Jun N-terminal kinase 1; *VEGFA*: vascular endothelial growth factor A.

^a^Housekeeping gene

## Data Availability

The data used to support the findings of this study are included within the article.
